# Long-term benefits to psychological health and well-being after ceremonial use of Ayahuasca in Middle Eastern and North African immigrants and refugees

**DOI:** 10.3389/fpsyt.2024.1279887

**Published:** 2024-04-10

**Authors:** Matthew X. Lowe, Hannes Kettner, Del R. P. Jolly, Robin L. Carhart-Harris, Heather Jackson

**Affiliations:** ^1^ Unlimited Sciences, Colorado Springs, CO, United States; ^2^ Psychedelics Division, Neuroscape, University of California, San Francisco, San Francisco, CA, United States; ^3^ Centre for Psychedelic Research, Imperial College London, London, United Kingdom

**Keywords:** refugee, MENA, ayahuasca, immigrant, psychedelics, mental health, longitudinal, naturalistic

## Abstract

**Background:**

Refugees and immigrants can experience complex stressors from the process of immigration that can have lasting and severe long-term mental health consequences. Experiences after ayahuasca ingestion are shown to produce positive effects on psychological wellbeing and mental health, including anecdotal reports of improved symptoms of trauma and related disorders. However, data on the longitudinal health impact of naturalistic ayahuasca use in Middle Eastern and North African (MENA) immigrant and refugee populations is limited.

**Aims:**

The current longitudinal online survey study was conducted to gather prospective data on ceremonial ayahuasca use in a group (N = 15) of primarily female MENA immigrants and refugees and to provide further insight into the patterns and outcomes surrounding that use. The study sought to assess self-reported changes in physical and mental health, well-being, and psychological functioning, examine relationships between aspects of individual mindset (e.g., psychedelic preparedness) prior to ayahuasca use and observed outcomes during (e.g., subjective drug effects) and afterwards (i.e., persisting effects), characterize risks and negative experiences, and describe trauma exposure and personal history.

**Results/Outcomes:**

Our findings revealed ceremonial use of ayahuasca is associated with significant improvements in mental health, well-being, and psychological functioning, including reductions in depression, anxiety, and shame, and increases in cognitive reappraisal and self-compassion. Most participants reported no lasting adverse effects and experienced notable positive behavioral changes persisting months after ingestion.

**Conclusion/Interpretation:**

While preliminary, results suggest naturalistic ayahuasca use might hold therapeutic potential for MENA populations exposed to trauma prior to and during the process of migration.

## Introduction

Individuals of Middle Eastern and North African (MENA) descent in the United States have been historically understudied (1). The MENA region spans the northern strip of Africa and the southwestern point of Asia, including Algeria, Bahrain, Comoros Islands, Djibouti, Egypt, Iran, Iraq, Israel, Jordan, Kuwait, Lebanon, Libya, Mauritania, Morocco, Oman, Palestine, Qatar, Saudi Arabia, Sudan, Tunisia, Egypt, Iraq, and Syria. There is an urgent need to attend to MENA individuals’ experiences of cumulative trauma, particularly as it relates to the process of immigration or refugee resettlement. Trauma not uncommonly experienced by immigrants and refugees includes complex stressors from the process of immigration, such as witnessing death or injury, traumatic exposure to physical and sexual violence, persecution, experiences of war and torture as civilians or soldiers, life threatening situations, and traumatic journeys prior to and during the process of migration, including separation from family, loss of home and livelihood, and acculturation problems (2–6).

Postmigration challenges tied to resettlement in another country pose a significant risk for poor mental health and psychological wellbeing and are often compounded by the loss of extended family, kinship, and sense of community (7). For new immigrants and refugees, psychological distress related to real and immediate dangers to family and friends left behind in situations of war, violence, and political upheaval is particularly salient (8). While MENA immigrants tend to have higher education levels than other immigrant groups, they also have a lower likelihood of being employed, and a lower income level (1). These challenges are particularly pronounced among MENA women, who face gender-based trauma and challenges and participate in the US workforce at a far lower rate than MENA men (9). Newly arrived refugees and immigrants generally have access to educational and employment opportunities in the United States, but several factors impede MENA women from taking advantage of such opportunities, including gender disparity affecting education inequality and language skills, gendered and familial issues, cultural expectations, labor market expectations, and personal wellbeing. For immigrant or refugee families, parental exposure to trauma during the process of migration may also lead children of foreign-born parents to face a wide spectrum of physical and psychological barriers that can impact psychological health and wellbeing (10). These experiences can have lasting and severe long-term mental health consequences, yet noncitizen immigrants and refugees face significant barriers to healthcare and mental health (11). To develop effective interventions, it is important to identify and build upon protective factors that improve psychological wellbeing for MENA Americans (1).

Ayahuasca, roughly translating to “vine of the soul” in the Quechua language (12), is a natural plant-based psychoactive brew, traditionally used in ceremonies throughout the Western Amazon basin (13). Ayahuasca is usually prepared by boiling stems of the *Banisteriopsis caapi* vine, which acts as a reversible monoamine oxidase inhibitor (MAOI), with leaves from the *Psychotria viridis* shrub or *Diplopterys cabrerana*, which contain the psychedelic tryptamine N,N-dimethyltryptamine (DMT) (14). The ayahuasca experience can produce significant alterations to one’s sense of self and reality, cognitive and emotional processing, and spatiotemporal orientation (15) and is known to be relatively safe to use (16). Historically, the use of ayahuasca in indigenous contexts is related to spiritual, social, cultural, and healing purposes (17). religious use of ayahuasca has been reported by diverse indigenous populations of the Amazon, and since the 1930s, several syncretic religious groups have adopted ayahuasca as a sacrament, including the Santo Daime, and UDV (União do Vegetal) ayahuasca religions (18). Although its use originated among indigenous communities throughout the Amazon basin, ayahuasca use in Western society has proliferated in recent years. In the Amazonian tradition, the ayahuasca ceremony is heavily influenced by a shaman or curandero, who often leads the ceremony and induces changes in the visionary and bodily experiences of participants (19). Shamanic transmission of knowledge is carried by lineages of believers, leading to heterogeneous practices across cultures.

Growing evidence suggests ayahuasca consumption may provide therapeutic relief in relation to treatment-resistant depression (20), trauma (21), suicidality (22), and grief (23). Somatic effects of ayahuasca are often strongly associated with psychotherapeutic processes of healing (24–27), and have been likened to intense psychotherapy (28) leading ayahuasca to have strong potential as a treatment for post-traumatic stress disorder and other trauma relatable disorders (29). Specifically, ayahuasca is associated with improvements in well-being and quality of life (30), and qualitative reports indicate greater self-love and compassion after use of ayahuasca (31). Data on the public health impact of naturalistic ceremonial ayahuasca use is limited, fueling concerns about rising ayahuasca use and widespread popular media coverage that may not accurately reflect evidence-based evaluation (32). Despite these concerns, preliminary epidemiological studies with large samples indicate no impairments in mental, physical, or social health following ayahuasca use and possible benefits for individual and collective health (33).

The current longitudinal online survey study was conducted to gather prospective data on ceremonial ayahuasca use in a group of primarily female MENA immigrants and refugees and to provide further insight into the patterns and outcomes surrounding that use. Specifically, the study aims were to: 1) prospectively assess self-reported changes in physical and mental health, well-being, and psychological functioning from before to after ceremonial ayahuasca use; 2) examine relationships between aspects of individual mindset (e.g., psychedelic preparedness) prior to ceremonial ayahuasca use and observed outcomes during (e.g., subjective drug effects) and afterwards (i.e., persisting effects); 3) characterize risks and negative experiences during and after ceremonial use of ayahuasca, and 4) describe trauma exposure and personal history of an immigrant and refugee community.

We tested *a priori* hypotheses that were informed by prior research. First, we hypothesized respondents would exhibit improvements in mental health, well-being, and psychological functioning from before to after ayahuasca use, consistent with prior evidence (33–35). Second, following previous research that individual traits predict response to psychedelics, such as absorption (36), psychological insight (37), and state of surrender (38), we hypothesized that these aspects of individual mindset (i.e., absorption, psychological insight, and state of surrender) measured before the experience would be significantly associated with subjective drug effects measured after the experience (i.e., mystical and challenging experiences, emotional breakthrough, and awe).

## Methods

### Study design

This prospective, naturalistic survey study enrolled mostly female participants of Middle Eastern or North African (MENA) origin identifying as an immigrant, refugee, or nonimmigrant with foreign-born parents. Participants included English-speaking adults aged 18 years or older planning to take ayahuasca outside clinical research settings in a group-based ceremony. Recruitment took place independently through an organizer of the ceremony not affiliated with the study. All participants attended the same retreat where the ceremony took place over five days, including two consecutive days of ayahuasca ingestion. On day one, arrival and introductions took place, followed by yoga and meditation sessions on day two. On day three, the day of the first ceremony, participants took part in breathwork and a sound bath. Immediately prior to the ceremony, participants reported smudging (ceremonial use of sage to clear out negative/stake energies), breathwork exercises, and a ritual honoring of “Pachamama” (Mother Earth). Participants reported ayahuasca was administered in a group setting over two consecutive days by a ceremonial guide at approximately 7:00 pm each day. Integration sessions immediately followed each ceremony and took place again in the morning following the ceremony and prior to departure Participants were provided with detailed instructions on clothing and materials allowed at the ceremony, and dietary and spiritual suggestions included a regimented diet beginning up to 14 days before the ceremony with a list of supplements and prescription medicines that can have contraindications with ayahuasca (See **Supplementary Materials**).

The study design was adapted and modified from Nayak and colleagues (39), consisting of six sequential web-based surveys assessing variables such as demographics, lifestyle, mindset, and personality traits, as well as characteristics of the experience itself such as dosage, ingestion method, intention, and setting, that could influence long-term effects and outcomes. Longitudinal measures were assessed before and after the ayahuasca sessions and were administered through Qualtrics XM secure online platform. The study was approved by an Institutional Review Board at the Western Institutional Review Board Copernicus Group (WCG IRB). Following an initial informed consent and demographics survey, participants completed 5 surveys with timing relative to the reference ayahuasca experience: 2 weeks before, same day prior to the ceremony, 1 to 3 days after, 2-4 weeks after, and 3-4 months after. Responses were collected from July 9, 2022, to November 29, 2022. In each of the 5 surveys, several open-ended questions were used to assess participants’ experiences throughout the duration of the study, and these qualitative results will be analyzed and reported in a companion study.

### Dosing

Ayahuasca was administered in the form of a brew containing leaves of *Psychotria Viridis* (the source of DMT) and the ayahuasca vine, *Banisteriopsis Caapi* (the source of enzymatic inhibitors of DMT metabolism). Traditional ayahuasca is variable in appearance, taste, and effects, with considerable variations in alkaloid profiles of ayahuasca from different sources (40). Due to these variations, approximate dosing information is provided for reference only, and a compound analysis was not performed. Dosage was measured in ounces, with approximately 0.5 ounces (14.8 milliliters) corresponding to “small”, 1.0 ounces (29.6 milliliters) corresponding to “medium”, and 2.0 ounces (59.2 milliliters) corresponding to “large”. No other substances were taken in conjunction with ayahuasca.

### Survey 1: consent and demographic information

Participants were invited to participate in the study if they 1) were at least 18 years old; 2) were able to read and write English fluently; 3) did not experienced a traumatic brain injury (TBI); 4) were planning an ayahuasca experience; and 5) were willing to complete baseline, pre-session, post-session, and follow-up surveys, and share an email address where they received reminders with links to survey assessments. Participants reviewed a waiver of documentation of informed consent explaining the study procedures, confirmed inclusion criteria, and provided basic demographic information including age, gender, race/ethnicity, education, and mental health history. Participants also recorded the purpose and intended date of the planned ayahuasca experience. An email address was provided where subsequent surveys and reminders would be sent. The estimated completion time of Survey 1 was 17 minutes.

### Survey 2: baseline 2 weeks pre-ceremony

A series of assessments were administered longitudinally in this survey, at baseline, again in the 2-4 week follow-up, and 3-4 month follow-up surveys. Primary outcomes included a modified 20-item Beck Depression Inventory II (BDI-II) to assess depressed mood (excluding an item about current suicidality due to lack of ability to respond adequately to potential imminent risk) (41) and the validated 20-item Short State-Trait Anxiety Inventory (STAI) assessing state (current) and trait (general) anxiety (42, 43). Secondary outcomes included the 10-item Emotion Regulation Questionnaire (ERQ) assessing cognitive reappraisal (i.e., ability to view emotional stimuli in a variety of ways) and expressive suppression (i.e., tendency to suppress emotional response in a given context) as two dimensions of emotion regulation (44); the 12-item Self-Compassion Scale – Short form (SCS-SF) was used to assess participants’ capacity for self-compassion, or the ability to hold one’s feelings of suffering with a sense of warmth, connection, and concerns (45); the 8-item External and Internal Shame Scale (EISS) was administered to assess external shame, which is shame focused on the experience of the self as seen in a judgmental way by others, and internal shame, which refers to self -focused negative evaluations and feelings about the self (46); the 12-item Cognitive Flexibility Scale (CFS) assessing self-reported ability to think and behave adaptively (47); the 4-item Patient-Reported Outcomes Measurement Information System Global Health (PROMIS-GH) physical health subscale assessing self-reported physical health (48); the 12-item Functional Assessment of Chronic Illness Therapy Spiritual Well-Being (FACIT-Sp) assessing spiritual well-being dimensions of faith, meaning, and peace (49); and the 13-item Copenhagen Burnout Inventory (CBI) assessing personal and work-related burnout and emotional exhaustion (50). Finally, the 44-item Big Five Inventory (BFI) assessed five major dimensions of personality: Openness, Conscientiousness, Extraversion, Agreeableness, and Neuroticism at 2 weeks prior and 3-4 months after the planned ayahuasca experience (51).

Additionally, some measures were collected only once before the dosing session, including drug use history, the 34-item Tellegen Absorption Scale (TAS) assessing openness to altered states (52), the 14-item Adverse Childhood Experience (ACE) scale (revised) assessing history of childhood physical and emotional abuse or neglect (53), and the single-item PTSD screener (SIPS) (54). The estimated completion time of Survey 2 was 67 minutes.

### Survey 3: same day prior to ceremony

Subjects were asked their planned dosage, intent of the session, outlook regarding the session, as well as physical indications such as current ailments, diet, sleep quality, and substance use.

Participants were administered the 14-item Psychedelic Predictor Scale (PPS) to capture thoughts and expectations right before the experience and the willingness to surrender to the experience across two subscales of set and setting (55). Finally, the 10-item state of surrender (SoS) scale was administered assessing level of psychological surrender or preoccupation before the session, which have previously shown correlations to mystical and challenging subjective effects of psilocybin, respectively (38). The estimated completion time of Survey 3 was 10 minutes.

### Survey 4: 1 to 3 days after ceremony

Survey 4 was completed 1-3 days after the second, and final, ayahuasca ceremony. Participants were asked the estimated dosage of ayahuasca they ingested. In addition, participants completed measures of the subjective qualities of the psychedelic experience. The 30-item Mystical Experience Questionnaire (MEQ30) was used to assess the degree of mystical-type (i.e., unitive, positive mood; transcending space or time, ineffable) qualities of the psychedelic experience, with scores **≥** 60% of the maximum score on each of the 4 subscales indicating a “complete mystical experience” (56). The 26-item Challenging Experience Questionnaire (CEQ) assessed a variety of difficult experiences that could arise during the ayahuasca session, comprised of seven factors: grief, fear, death, insanity, isolation, physical distress, and paranoia (57). The 30-item Awe Experience Scale (AWE-S) was administered to assess the complex emotion of awe and comprised a 6-factor structure including altered time perception, self-diminishment, connectedness, perceived vastness, physical sensations, and need for accommodation (58). The 6-item Emotional Breakthrough Inventory (EBI) was used as a measure of emotional release/breakthrough experienced during an acute psychedelic state (59). The 6-item Psychological Insight Scale (PIS) was used to assess psychological insight and accompanied behavioral changes after a psychedelic experience (37). An adapted 8-item version of the Communitas Scale (CS) was used to assess acute relational experiences of perceived togetherness and shared humanity to investigate psychosocial mechanisms pertinent to psychedelic ceremonies and retreats (60). The estimated completion time of Survey 4 was 35 minutes.

### Surveys 5 and 6: follow-ups at 2-4 weeks and 3-4 months post-ceremony

In surveys 5 and 6, completed approximately 2-4 weeks and 3-4 months after the ayahuasca ceremony, respectively, participants were asked to rate the meaningfulness, insightfulness, and spiritual significance of the experience. Items asked: “How personally meaningful/psychologically insightful/spiritually significant was your ayahuasca experience and your contemplation of that experience?” Responses ranged across eight options from “No more than routine, everyday experiences” to “The single most meaningful/insightful/spiritually significant experience of my life.” As noted above, the BFI was re-administered in survey 6 at 3-4 months after the ayahuasca experience. Otherwise, all longitudinal measures (i.e., BDI-II, STAI, ERQ, CFS, PROMIS-GH, FACIT-Sp, CBI) were re-administered in these surveys. The estimated completion time of Survey 5 and 6 was 48 minutes.

### Data analysis

Descriptive statistics including means, SD and ranges were performed for demographic variables. Measures were included in the analysis if they were completed by ≥ 60% of participants at each timepoint. For longitudinal measures, Bayesian inference was used to compare differences across time points. Bayes factors (BF) indicate the relative strength of evidence for two theories (e.g., 61, 62). The Bayes factor B comparing an alternative hypothesis to the null hypothesis means that the data are B times more likely under the alternative than under the null. Following Jeffreys et al. (63), a conventional cut-off for substantial evidence to support the alternate hypothesis includes a Bayes factor greater than 3. Conversely, anything between 1/3 and 3 is considered weak or “anecdotal” evidence. However, to account for adjustments required to control type I error rates, a suggested cut-off score was set at 2 (64). Comparisons for longitudinal outcomes (i.e., BDI-II, STAI, ERQ, CFS, PROMIS-GH, FACIT-Sp, CBI) were performed using a Bayesian paired samples t-test (BF_10_) using JASP statistical software (65) for each variable pair across time (*baseline* versus *2-4 weeks*; *baseline* versus *3-4 months*; *2-4 weeks* versus *3-4 months*). For any comparisons equal to or greater than the suggested cutoff score of 2, standard paired-samples t-test were performed for confirmatory analyses, and effect sizes were reported using Cohen’s d and 95% confidence intervals. In addition to Bayesian inference, linear mixed models were included to assess overall significance of primary and secondary outcomes (see **Supplementary Materials**).

To assess what aspects of the psychedelic experience were associated with long-term changes on the primary outcomes, change scores on the BDI, STAI-T and STAI-S were calculated by subtracting baseline scores from each of the endpoints (2-4 weeks and 3-4 months). Subsequently, Pearson correlations were computed between the resulting change scores and each of the five post-session measures MEQ, CEQ, EBI, AWE-S, COMS, resulting in 10 comparisons per outcome variable: five for the 2-4 week and five for the 3-4 month endpoint.

*A priori* correlations (Pearson’s r) assessed associations between several measures assessed prior to the ingestion of ayahuasca, including absorption (TAS), openness to surrendering to the experience (SoS), and set and setting (PPS) with outcomes of mystical experiences (MEQ30), emotional breakthrough (EBI), psychological insight (PIS) and awe (AWE-S). Baseline adverse childhood experiences (ACE) were also correlated with post-session challenging experiences (CEQ).

## Results

### Participant demographics

Fifteen participants provided informed consent for the study (Survey 1). Sample sizes for each of the following surveys were N = 14 (Survey 2; weeks pre-session), N = 15 (Survey 3; same day prior pre-session), N = 15 (Survey 4; 1-3 days post-session), N = 14 (Survey 5; 2-4 weeks post-session), and N = 15 (Survey 6; 2-3 months post-session). Mean age (SD) was 30.1 years (7.2). Most participants were female (n = 11; 73.3%), residing in the United States (n = 11; 73.3%), held a bachelor’s level or higher degree (n = 11; 73.3%), and classified themselves as religious (n = 9; 60.0%). Participants identified as an immigrant or refugee (n = 10; 66.7%), nonimmigrant with foreign-born parents (n = 3; 20%), or nonimmigrant visitor primarily residing in the country of their birth (n = 2; 13.3%). Participants identified as Arab, Middle Eastern, or North African (n = 12; 80.0%) and Mixed Race (n = 3; 20.0%), and all participants spoke Arabic and English.

### Personal history

In Survey 2, while most participants (n = 9; 60%) reported previously taking a dose of a classic psychedelic (e.g., psilocybin mushrooms, psilocybin, LSD, ayahuasca, mescaline, DMT, etc.) that produced moderate to strong psychoactive effects, most participants had no experience of using ayahuasca prior to enrolling in the study (n = 12; 80.0%). The most commonly reported prior psychedelic use was psilocybin (n = 8; 53.3%), followed by LSD (n = 6; 40.0%). Most participants (n = 12; 80.0%) had been previously diagnosed or struggled with a mental health condition, including an anxiety disorder (n = 10; 66.7%), mood disorder (n = 8; 53.3%), eating disorder (n = 4; 26.7%), personality disorder (n = 1; 6.7%), or substance-related disorder (n = 1; 6.7%).

### Experiences of trauma

In Survey 2, using the single-item PTSD screener (SIPS), 50% of the sample (n = 7) reported that they had been recently bothered by an experience that caused them to believe they would be injured or killed. Most participants (n = 11; 78.6%) reported that they had experienced, witnessed, or had been repeatedly confronted with a traumatic experience. Forms of trauma experienced by participants included sexual assault (n = 6; 42.86%), child abuse (n = 6; 42.86%), physical assault (n = 4; 28.56%), serious accident (n = 2; 14.29%), natural disaster (n = 1; 7.14%), and life-threatening illness (n = 1; 7.14%). Some participants described their experience as other trauma (n = 5; 35.71%), including descriptions such as, “Leaving my home country for the last time where I knew there was a threat to me and my partner”. Many participants (n = 7; 50.0%) reported this experience involved actual or threatened death, serious injury, or sexual violence.

### Intention

On the day of the ayahuasca ceremony immediately prior to the experience (Survey 3), participants characterized the purpose for the ayahuasca ceremony as (non-exclusively) self-exploration (n = 13; 86.7%), creativity (n = 9; 60.0%), mental health (n = 8; 53.3%), physical health (n = 5; 33.3%), therapy (n = 4; 26.7%), productivity (n = 4; 26.7%), and recreation (n = 2; 13.3%). All respondents (n = 15; 100.0%) reported setting a specific intention for the experience. For example, one participant stated, “I want to heal from the abuse I endured to be able to be the best version of myself,” and another wrote, “I want to get back in touch with my true self. I want to be brave to become my authentic self publicly.”

### Physical indications, diet, and exercise

In the period leading up to the ceremony (Survey 2), most participants reported regular (≥ once/per week) physical exercise (n = 10; 71.4%), and three or more servings of fruit and vegetables each day (n = 8; 57.1%). On the day of the ceremony (Survey 3), most participants (n = 13; 86.7%) reported no physical ailments. In the 24 hours prior to the experience, mean (SD) reported restful sleep was 7.0 (1.5) hours, all participants reported no alcohol consumption or use of caffeine, cannabis, selective serotonin reuptake inhibitors (SSRIs), benzodiazepine, prescription or other stimulants, nootropic, opioid, or other psychedelic, and one participant reported use of nicotine (n = 1; 6.7%). In the 48 hours prior to the experience, many participants (n = 7; 46.7%) reported no consumption of gluten, dairy, red meat, white meat, seafood, or processed sugars, and some reported consumption of gluten (n = 4; 26.7%), dairy (n = 2; 13.3%), white meat (n = 2; 13.3%), and seafood (n = 1; 6.7%).

### Dosage

The majority (n = 8; 53.3%) of participants reported taking more than one dose on each day of the ceremony. No other substances were taken in conjunction with ayahuasca. On day one, the mean (SD) dosage was 1.41 (0.73) ounces or 41.7 (21.6) milliliters, corresponding to a medium-to-large dose, and on day two, the mean (SD) dosage was 1.31 (0.53) ounces or, 38.7 (15.7) milliliters corresponding to a medium-to-large dose.

### Setting

Participants attended an ayahuasca ceremony as part of a five-day retreat. The ceremony took place indoors in a darkened room used as a sensory limiting tool. In addition to the ceremonial guide, three male helpers, three female helpers, a female licensed therapist, and a translator attended the ceremony. The ceremony guide reported receiving over a decade of training from a shaman in the Amazonian regions of Peru, and several years independently leading ayahuasca ceremonies. In the post-session survey completed 1 to 3 days post-ceremony (Survey 4; N = 15), participants reported live instrumentals, live vocals, and shamanic or ritualistic music were performed by the ceremony guide. In a Likert scale rating how important the ceremony and guide were in shaping their experience, most of the participants (n = 13; 86.7%) reported that both the guide and ceremony had a significant positive impact. In the morning immediately following both ayahuasca sessions, participants performed group integration practices and were also offered independent one-on-one sessions with a licensed therapist.

### Primary outcomes of longitudinal measures

#### Mood

**Figure 1:**
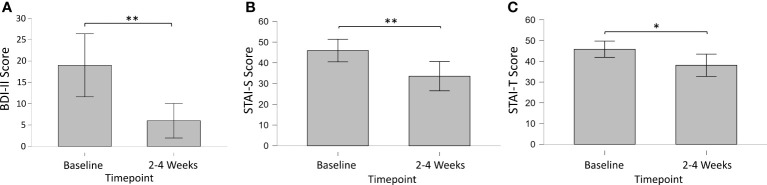
Longitudinal changes in primary outcomes measures of **(A)** Depression (BDI-II), **(B)** State (STAI-S) and **(C)** Trait (STAI-T) Anxiety from baseline to 2-4 weeks post-session. * = < 0.05; ** < 0.01.

In Survey 2 (2-4 Weeks Pre-Session), most participants (n = 13; 86.7%) indicated they had previously struggled with depressed mood. Modified BDI-II mean (SD) total scores of these participants were 19.0 (12.2) for survey 2, with 71.4% (n = 10) of respondents meeting criteria for some form of depression (mild mood disturbance). For Surveys 5 and 6, modified BDI-II mean (SD) total scores were 6.0 (6.0) and 12.2 (11.7), respectively, with 7.7% of respondents (n = 1) meeting depression criteria for Survey 5, and 38.5% (n = 5) meeting depression criteria for Survey 6. Bayesian analysis of BDI scores showed substantial evidence for a decrease in depression from baseline to 2-4 weeks post-session (BF_10_ = 21.72), but not from baseline to 3-4 months post-session (BF_10_ = 0.86), or from 2-4 weeks to 3-4 months post-session (BF_10_ = 1.45). A paired-sample t-test [Cohen’s d; 95% CI] confirmed significantly decreased depression from baseline to 2-4 weeks post-session (p = 0.002; t = 4.11; df = 10) with a large effect size [1.24; 0.43, 2.02] (**Figure 1**).

#### Anxiety

In Survey 2 (2-4 Weeks Pre-Session), most participants (n = 13; 86.7%) indicated they had previously struggled with anxiety. Short STAI state and trait anxiety mean (SD) total scores were 45.9 (12.0) and 45.9 (8.8), respectively. For Survey 2, 69.2% (n = 9) of respondents met criteria for high-risk state anxiety, and 76.9% (n = 10) met criteria for high-risk trait anxiety. For Survey 5, short STAI state and trait anxiety mean (SD) total scores were 33.6 (9.9) and 38.1 (10.2), respectively. For Survey 5, 22.2% (n = 2) of respondents met criteria for high-risk state anxiety, and 22.2% (n = 2) met criteria for high-risk trait anxiety. For Survey 6, short STAI state and trait anxiety mean (SD) total scores were 37.0 (11.7) and 39.6 (10.3), respectively. For Survey 6, 36.4% (n = 4) of respondents met criteria for high-risk state anxiety, and 45.5% (n = 5) met criteria for high-risk trait anxiety. Bayesian analysis of STAI scores showed substantial evidence for a decrease in state anxiety from baseline to 2-4 weeks post-session (BF_10_ = 7.07), but not from baseline to 3-4 months post-session (BF_10_ = 0.68), or from 2-4 weeks to 3-4 months post-session (BF_10_ = 0.36). A paired-sample t-test [Cohen’s d; 95% CI] confirmed significantly decreased state anxiety from baseline to 2-4 weeks post-session (p = 0.009; t = 3.45; df = 8) with a large effect size [1.15; 0.27, 1.98]. Bayesian analysis of STAI scores showed substantial evidence for a decrease in trait anxiety from baseline to 2-4 weeks post-session (BF_10_ = 3.02), but not from baseline to 3-4 months post-session (BF_10_ = 0.63), or from 2-4 weeks to 3-4 months post-session (BF_10_ = 0.36). A paired-sample t-test [Cohen’s d; 95% CI] confirmed significantly decreased trait anxiety from baseline to 2-4 weeks post-session (p = 0.026; t = 2.73; df = 8) with a large effect size [0.91; 0.10, 1.68].

### Secondary outcomes of longitudinal measures

#### Emotion regulation

**Figure 2:**
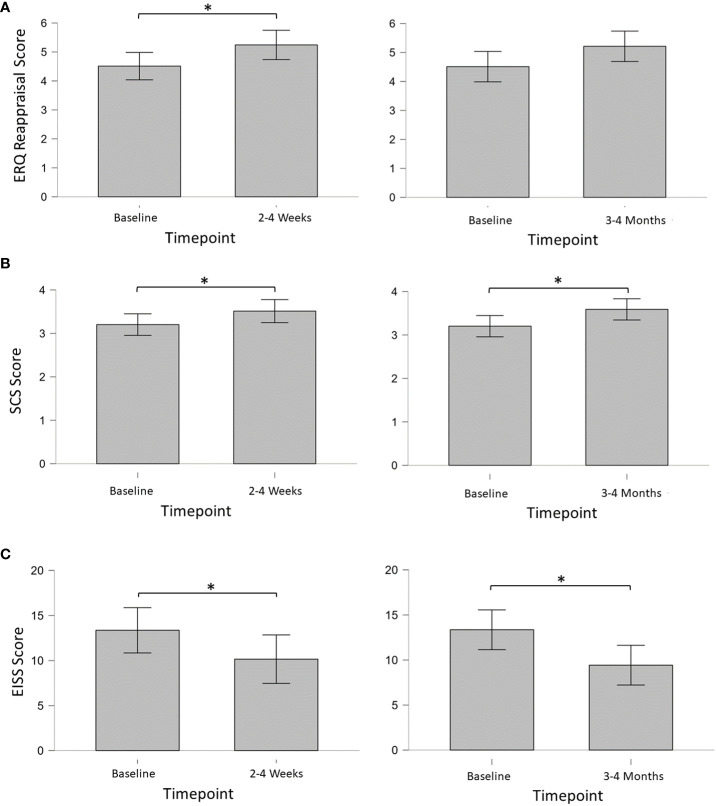
**(A)** Mean Emotion Regulation Questionnaire (ERQ) Cognitive Reappraisal scores; **(B)** mean Self-compassion Scale – Short Form (SCS-SF) scores; **(C)** total mean External and Internal Shame Scale (EISS) scores. * = < 0.05.

Mean (SD) ERQ cognitive reappraisal and expressive suppression scores on Survey 2 were 4.5 (1.1) and 3.7 (0.8), respectively (**Figure 2**). For Survey 5, ERQ cognitive reappraisal and expressive suppression scores were 5.2 (1.3) and 4.2 (1.2), respectively. For Survey 6, ERQ cognitive reappraisal and expressive suppression scores were 5.2 (1.3) and 3.6 (1.6), respectively. Bayesian analysis of ERQ scores showed weak evidence for an increase in cognitive reappraisal from baseline to 2-4 weeks post-session (BF_10_ = 2.02), but not from baseline to 3-4 months post-session (BF_10_ = 1.34), or from 2-4 weeks to 3-4 months post-session (BF_10_ = 0.30). A paired-sample t-test [Cohen’s d; 95% CI] confirmed significantly increased cognitive reappraisal from baseline to 2-4 weeks post-session (p = 0.037; t = -2.34; df = 12) with a medium effect size [-0.65; 1.24, -0.04]. Bayesian analysis revealed limited to no evidence for differences at any timepoint for expressive suppression.

#### Self-compassion

The mean (SD) SCS-SF score on Survey 2 was 3.2 (0.6), indicating a moderate level of self-compassion. Mean (SD) scores for Survey 5 and Survey 6 were 3.5 (0.7) and 3.6 (0.7), respectively, indicating a high level of self-compassion. Bayesian analysis of SCS-SF scores showed weak evidence for an increase in self-compassion from baseline to 2-4 weeks post-session (BF_10_ = 2.40), and from baseline to 3-4 months post-session (BF_10_ = 2.28). No evidence of change was found from 2-4 weeks to 3-4 months post-session (BF_10_ = 0.28). Paired-samples t-tests [Cohen’s d; 95% CI] of SCS-SF scores confirmed significantly increased self-compassion from baseline to 2-4 weeks post-session (p = 0.030; t = -2.46; df = 12) with a medium effect size [-0.68; -1.28, -0.06], and from baseline to 3-4 months (p = 0.031; t = -2.42; df = 13) with a medium effect size [-0.65; -1.22, -0.06].

#### Shame

Mean (SD) total EISS scores on Surveys 2, 5, and 6 were 13.4 (5.9), 10.2 (5.5), and 9.4 (4.6), respectively. Bayesian analysis of EISS scores showed weak evidence for a decrease in shame from baseline to 2-4 weeks post-session (BF_10_ = 2.14), and substantial evidence for a decrease in shame from baseline to 3-4 months post-session (BF_10_ = 3.62). No evidence was found from 2-4 weeks to 3-4 months post-session (BF_10_ = 0.28). Paired-samples t-tests [Cohen’s d; 95% CI] of EISS scores confirmed significantly decreased shame from baseline to 2-4 weeks post-session (p = 0.035; t = -2.34; df = 12) with a medium effect size [0.66; 0.05, 1.25], and from baseline to 3-4 months (p = 0.017; t = 2.72; df = 13) with a medium effect size [0.73; 0.12, 1.31].

#### Non-significant and excluded secondary outcomes

No evidence for longitudinal changes were found across timepoints for the FACIT-Sp (all ps ≥ 0.097), CFS (all ps ≥ 0.222), PROMIS-GH (all ps ≥ 0.297), and BFI (all ps ≥ 0.095). The CBI was excluded from analysis due to insufficient completion rates (≤ 60%).

### Associations of longitudinal changes and acute psychedelic effects

#### Changes in depression

**Figure 3:**
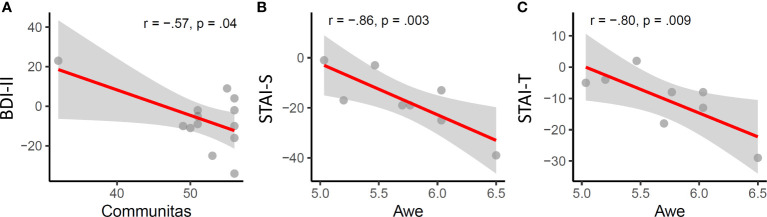
Acute psychedelic effects of communitas and awe are significantly associated with longitudinal changes in depression **(A)** state **(B)** and trait anxiety **(C)**.

Pearson correlations between change scores on the BDI from baseline to the 2-4 week and 3-4 months endpoints versus each of the five post-session measures of acute psychedelic effects were calculated. Among the 10 correlations, the only significant association was found between communitas scores and depression changes between baseline and the follow-up endpoint at 3-4 months (r = .57, p = .04, **Figure 3**). However, this association would not have survived correction for multiple comparisons.

#### Changes in anxiety

Among the five measures of acute subjective effects, only awe was significantly associated with changes in anxiety, specifically with state (r = -.86, p = .003) and trait anxiety (r = -.8, p = .009) at the 2-4 week endpoint.

### Associations of acute psychedelic effects and pre-session measures

#### Challenging experiences

The mean (SD) total Challenging Experience Questionnaire (CEQ) score was 0.38 (0.26) in Survey 4. CEQ scores were not significantly associated (Pearson’s r) with pre-session Adverse Childhood Experience (ACE) scores in Survey 2 (r = 0.132, p = 0.716).

#### Mystical experiences

The mean (SD) total Mystical Experience Questionnaire (MEQ30) score in Survey 4 was 0.75 (0.15), with 9 (60.0%) meeting *a priori* criteria for a “complete mystical experience.” MEQ30 scores were significantly associated (Pearson’s r) with pre-session Psychedelic Predictor Scale (PPS) scores of Set (r = 0.627, p = 0.012) and Setting (r = 0.607, p = 0.016) in Survey 3 and with pre-session Tellegen Absorption Scale (TAS) scores (r = 0.576, p = 0.031) in Survey 2. MEQ30 scores were not significantly associated with State of Surrender (SoS) scores in Survey 2 (r = 0.490, p = 0.064).

#### Emotional breakthrough

The mean (SD) total Emotional Breakthrough Inventory (EBI) score was 0.65 (0.22) Survey 4. EBI scores were significantly associated (Pearson’s r) with pre-session PPS scores of Set (r = 795, p < 0.001) but not Setting (r = 0.432, p = 0.108) in Survey 3, and were not significantly associated (Pearson’s r) with pre-session TAS scores (r = 0.531, p = 0.051) in Survey 2. EBI scores were not significantly associated with SoS scores in Survey 2 (r = 0.403, p = 0.137).

#### Psychological insight

The mean (SD) total Psychological Insight Scale (PIS) score was 0.72 (0.25) Survey 4. PIS scores in Survey 4 were significantly associated (Pearson’s r) with pre-session PPS scores of Set (r = 0.650, p = 0.009) but not Setting (r = 0.305, p = 0.268) in Survey 3, and were significantly associated with SoS scores in Survey 2 (r = 0.702, p = 0.004). PIS scores were not significantly associated (Pearson’s r) with pre-session TAS scores (r = 0.342, p = 0.231) in Survey 2.

#### Awe

The mean (SD) total Awe Experience Scale (AWE-s) score was 0.77 (0.88) in Survey 4. AWE-S scores were significantly associated (Pearson’s r) with pre-session PPS scores of Set (r = 0.739, p = 0.002) but not Setting (r = 0.305, p = 0.268) in Survey 3, and were significantly associated (Pearson’s r) with pre-session TAS scores (r = 0.639, p = 0.014) in Survey 2. AWE-S scores were not significantly associated with SoS scores in Survey 2 (r = 0.379, p = 0.164).

#### Communitas

The mean (SD) total Communitas Scale (CS) score was 0.91 (0.11) in Survey 4. CS scores were significantly associated with pre-session PPS scores of Setting (r = 0.540, p = 0.038), but not Set. CS scores were not significantly associated with either TAS or SoS scores.

## Retrospective measures of change

### Rating the overall experience

In Survey 4 (1-3 Days Post-session), on a 7-point Likert scale rating the experience ranging from “Extremely negative” to “Extremely positive”, most participants rated the experience as positive (n = 14; 93.3%), and the majority rated the experience as “Extremely positive” (n = 12; 80.0%). No participants rated the experience as negative, and one participant (n = 1; 7%) rated the experience as “Neither positive nor negative”.

### Attributions of meaning, spiritual significance, insight, and psychological challenge

In Survey 5 (2-4 weeks post-session), most participants considered the ceremony to be among the top 10 most personally meaningful (n = 13; 92.9%), top 10 most spiritually meaningful (n = 12; 85.7%), and top 10 most psychologically insightful (n = 12; 85.7%) experiences of their lives (**Table 1**). Many respondents (n = 6; 42.9%) considered the ceremony as among the top 10 most psychologically challenging experiences of their life. In Survey 6 (3-4 Months post-session), most participants still considered the ceremony to be among the top 10 most personally meaningful (n = 9; 60.0%), top 10 most spiritually meaningful (n = 9; 60.0%), and top 10 most psychologically insightful (n = 9; 60.0%) experiences of their lives. Many respondents (n = 7; 46.7%) reported that the ceremony was among the top 10 most psychologically challenging experiences of their lives.

**Table 1:** Retrospective ratings on ayahuasca experience and subsequent use (Surveys 5 and 6).

	2-4 weeks, mean (SD)	3-4 months, mean (SD)
**How personally meaningful was your ayahuasca experience and your contemplation of that experience? ^1^**	6.7 (1.0)	5.3 (2.4)
**How spiritually significant was your ayahuasca experience and your contemplation of that experience? ^1^**	6.8 (1.1)	5.5 (2.4)
**How personally psychologically insightful was your ayahuasca experience and your contemplation of that experience? ^1^**	6.8 (1.3)	5.4 (2.4)
**How psychologically challenging was the most psychologically challenging portion of the ayahuasca experience? ^1^**	5.3 (2.3)	4.8 (2.5)
**Do you believe that the ayahuasca experience and your contemplation of that experience has led to long-term and persisting changes in your current sense of personal well-being or life satisfaction? ^1^**	6.4 (0.8)	6.1 (1.1)
**Have you experienced any persisting negative effects from your ayahuasca experience, which lasted beyond the duration of the drug’s effects?** ^2^	**2-4 weeks,** **n (%)**	**3-4 months,** **n (%)**
None	9 (64.3)	9 (60.0)
Mood fluctuations	1 (7.1)	3 (20.0)
Confusion	3 (21.4)	0 (0.0)
Loneliness	0 (0.0)	4 (26.7)
Lowered motivation	0 (0.0)	3 (20.0)
Depressive notions	0 (0.0)	2 (13.3)
Persisting hallucinations	1 (7.1)	0 (0.0)
**Have you experienced any notable behavioral changes since this ayahuasca session?**	**2-4 weeks,** **n (%)**	**3-4 months,** **n (%)**
Reduced or stopped using other drugs	6 (42.9)	2 (13.3)
Started using other drugs more often/heavily	1 (7.1)	1 (6.7)
Reduced craving or use of alcohol	3 (21.4)	5 (33.3)
Increased craving or use of alcohol	0 (0.0)	0 (0.0)
Improved diet/nutrition	8 (57.1)	7 (46.7)
Worsened diet/nutrition	0 (0.0)	1 (6.7)
Increased physical activity/exercise	5 (35.7)	7 (46.7)
Decreased physical activity/exercise	1 (7.1)	2 (13.3)
Improved relationships with others	9 (64.3)	7 (46.7)
Worsened relationships with others	0 (0.0)	0 (0.0)
Improvements in career/work life	8 (57.1)	4 (26.7)
Worsening of career/work life	0 (0.0)	1 (6.7)
None of these	2 (14.3)	2 (13.3)

^1^ Ratings provided on the following 8-point scale: No more than routine, everyday personally meaningful/spiritually significant/psychologically insightful/challenging experiences=1; Similar to experiences that occur on average once or more a week=2; Similar to experiences that occur on average once a month=3; Similar to experiences that occur on average once a year=4; Similar to experiences that occur on average once every 5 years=5; Among the 10 most personally meaningful/spiritually significant/psychologically insightful/challenging experiences of my life=6; Among the 5 most personally meaningful/spiritually significant/psychologically insightful/challenging experiences of my life=7; The single most personally meaningful/spiritually significant/psychologically insightful/challenging experience of my life=8.

^2^ Ratings provided on the following 7-point scale: Strong positive change that I consider desirable=1; Moderate positive change that I consider desirable=2; Slight positive change that I consider desirable=3; No change=4; Slight negative change that I consider undesirable=5; Moderate negative change that I consider undesirable=6; Strong negative change that I consider undesirable=7.

### Self-reported behavioral changes after ceremonial use of ayahuasca

85.7% and 86.7% of participants reported notable behavioral changes at 2-4 weeks and 3-4 months after their ayahuasca experience, respectively (**Table 1**). The most commonly reported behavioral changes were improved relationships with others (n = 9; 64.3% at 2-4 weeks, and n = 7; 46.7% at 3-4 months), improved diet/nutrition (n = 8; 57.1% at 2-4 weeks, and n = 7; 46.7% at 3-4 months), improvements in career/work life (n = 8; 57.1% at 2-4 weeks, and n = 4; 26.7% at 3-4 months), increased physical activity/exercise (n = 5; 35.7% at 2-4 weeks, and n = 7; 46.7% at 3-4 months), reduced or stopped using other drugs (n = 6; 42.9% at 2-4 weeks, and n = 2; 13.3% at 3-4 months), and reduced craving or use of alcohol (n = 3; 21.4% at 2-4 weeks, and n = 5; 33.3% at 3-4 months). Almost all respondents characterized their experience using ayahuasca as beneficial 2-4 weeks (92.6%), and all at 3-4 months (100.0%) afterwards. When participants were asked if they believed the ayahuasca experience and their contemplation of that experience led to long-term and persisting changes in their current sense of personal well-being or life satisfaction, all participants considered the change as positive and desirable at 2-4 weeks, and 93.3% of considered the change as positive and desirable at 3-4 months. One participant (6.7%) reported negative and undesirable changes at 3-4 months.

### Symptoms & adverse effects

Participants reported several adverse events (**Table 2**) during and after the ayahuasca ceremony. While most adverse events did not persist in the 24 hours following the ceremony, respondents reported some persisting symptoms including lack of appetite (n = 6; 40.0%) and fatigue (n = 4; 26.7%). Some participants (n = 4; 26.7%) experienced physical pain during the ceremony, with a mean (SD) pain rating of 6.75 (0.50) on a scale out of 10, lasting a mean (SD) duration of 4.0 (1.6) hours. Descriptions of where pain sensation occurred included abdomen (stomach area) (n = 3; 20.0%), chest area (n = 2; 13.3%), anal/perineal/genital (n = 2; 13.3%), lower back/spine (n = 1; 6.7%) and shoulder (n = 1; 6.7%). No respondents reported seeking medical care during the experience, and many (n = 6; 40%) reported seeking psychological care. Descriptions of these events included speaking with the therapist, helpers, and other participants on site (e.g., “I talked to some of the helpers and participants and that helped me to integrate and feel less anxious or angry.”). Regarding persisting negative effects, most participants reported no persisting negative effects at each longitudinal follow up (Survey 5 & 6; n = 9; 60.0%). In the first longitudinal follow up (Survey 5; 2-4 Weeks Post-Session), persisting negatives effects included confusion (n = 3; 21.4%), disorientation (n = 1; 7.1%), persisting hallucinations (n = 1; 7.1%), mood fluctuations (n = 1; 7.1%), fear (n = 1; 7.1%), anger (n = 1; 7.1%), and headaches (n = 1; 7.1%). In the second longitudinal follow up (Survey 6; 3-4 Months), persisting negatives effects included loneliness (n = 3; 20.0%), depressive notions (n = 2; 13.3%), lowered motivation (n = 2; 13.3%), and mood fluctuations (n = 1; 6.7%).

**Table 2:** Symptoms during (in-session) and after (< 24 hours post-session) ayahuasca (Survey 4).

Adverse Event	In-session, n (%)	< 24 hours post-session, N (%)
Physical pain (That didn’t already exist)	4 (26.7)	0 (0.0)
Lack of appetite	8 (53.3)	6 (40.0)
Tremors	2 (13.3)	2 (13.3)
Nausea and/or vomiting	10 (66.7)	2 (13.3)
Tactile disturbances	0 (0.0)	1 (6.7)
Increased heart rate	2 (13.3)	0 (0.0)
Cramping	4 (26.7)	1 (6.7)
Headaches	6 (40.0)	3 (20.0)
Drowsiness	3 (20.0)	1 (6.7)
Depression or low mood	3 (20.0)	4 (26.7)
Confusion	5 (33.3)	2 (13.3)
Insomnia	4 (26.7)	4 (26.7)
Difficulty concentrating	2 (13.3)	2 (13.3)
Heart pounding, or sweating	2 (13.3)	0 (0.0)
Irritability	3 (20.0)	3 (20.0)
Restlessness	7 (46.7)	3 (20.0)
Hallucinations	10 (66.7)	2 (13.3)
Blurred vision	5 (33.3)	1 (6.7)
Seizures	0 (0.0)	0 (0.0)
Anxiety	4 (26.7)	0 (0.0)
Fever	3 (20.0)	0 (0.0)
Fatigue	5 (33.3)	5 (33.3)
Other (please describe)	4 (26.7)	2 (13.3)
None of the above	1 (6.7)	4 (26.7)

## Discussion

This study presents a prospective, longitudinal assessment of psychological health and wellbeing after ceremonial use of ayahuasca in a group of primarily female immigrants and refugees of Middle East and North Africa (MENA) descent. We report significant improvements in mental health, well-being, and psychological functioning, including reductions in depression, anxiety, and shame, and increases in cognitive reappraisal and self-compassion. Additionally, most participants reported positive behavioral changes which persisted for months following the ceremony, such as improved relationships, diet/nutrition, career/work life, increased physical activity/exercise, and reduced use or craving of alcohol and other drugs. Only a small minority of participants experienced lasting adverse effects such as confusion and mood fluctuations, suggesting monitored, ceremonial use of ayahuasca may represent a relatively low safety risk. Although preliminary, our findings suggest ceremonial ayahuasca use may hold broad therapeutic potential for MENA populations exposed to trauma prior to and during the process of migration.

### Stressor exposure and consequences in immigrants and refugees of MENA descent

Approximately 40% of the 60 million individuals displaced worldwide originate from MENA regions (66). There is a critical failure of international agreements to effectively provide viable solutions to the humanitarian ramifications of mass population movements, especially in vulnerable population groups such as immigrants and refugees, who have struggled to retain the rights, quality of life, and access to health care and educational standards (67). It has been well documented that accumulated lifetime trauma experience increases the likelihood of developing psychological distress and psychiatric disorders [e.g., (68)]. Compared with the general population, MENA immigrants and refugees may face increased rates of traumatic experiences, including historical trauma and pervasive institutional discrimination (1). A primary health risk for immigrants and refugees from the MENA regions stems from mental health disorders, mainly PTSD, depression, and anxiety (69). Due to limited access to mental health services, these individuals may neglect their conditions and subsequent consequences, often resulting in negative long-term social, psychological, and economic impacts (70). Consistent with these findings, nearly 80% of participants in the present study reported that they had experienced, witnessed, or had been repeatedly confronted with a traumatic experience, including sexual assault, child abuse, physical assault, serious accident, natural disaster, and life-threatening illness, for example, “Leaving my home country for the last time where I knew there was a threat to me and my partner.” Half of the participants reported these experiences involved actual or threatened death, injury, or sexual violence. These experiences can produce profound long-term consequences for mental health and wellbeing. In fact, over 80% of participants indicated that they had been previously diagnosed or struggled with depressed mood or anxiety, and over 70% of participants met the criteria for some form of depression and high-risk anxiety. The therapeutic processes associated with ayahuasca use may be particularly valuable for the cumulative racial-ethnic trauma experiences among immigrants and refugees of MENA descent, including the ability to reconceptualize and process traumatic experiences, which may in turn lead to improvements in mental health and wellbeing (71).

### Ceremonial ayahuasca use is associated with benefits to mental health

Accumulating evidence from cross-sectional, preclinical, and experimental studies suggest that ayahuasca has both antidepressant and anxiolytic effects (72). Robust evidence from controlled and open-label trials shows reduced depression scores following the administration of ayahuasca (20, 73, 74). Similarly, although anxiety studies show mixed results [see (33)], some studies suggest ayahuasca may have therapeutic potential in the treatment of anxiety [e.g., (75, 76)]. Consistent with these findings, results of the present study found significant reductions in the primary outcome measures of depression and anxiety 2-4 weeks after the ingestion of ayahuasca in a ceremonial setting. While more than two thirds of respondents met criteria for some form of depression and high-risk anxiety prior to the ceremony, only 7.7% and 22.2% of participants met criteria 2-4 weeks after ingestion of ayahuasca, respectively. However, significant reductions in scores of both depression and anxiety did not persist 3-4 months after ingestion. Although these findings suggest the antidepressant and anxiolytic effects of ceremonial ayahuasca use may have a time-limited window similar to observational and clinical trial data of psilocybin-assisted treatment (39, 77), other evidence supports sustained reductions in depression in clinically depressed patients 1 year after attendance of an ayahuasca ceremony (78). Further research is needed to examine the long-term efficacy of ayahuasca on mental health in different populations and broader samples, and whether some individuals may benefit from repeated use to sustain the antidepressant and anxiolytic effects of ayahuasca ingestion. Nevertheless, the present results suggest ceremonial use of ayahuasca may hold significant treatment potential for immigrants and refugees of MENA descent diagnosed or struggling with depression and anxiety. In the present study, our findings show that ayahuasca use administered in a monitored, ceremonial setting may represent a promising treatment strategy to address mental health and wellbeing challenges experienced by immigrants and refugees of MENA descent.

### Positive changes to experiences of shame, self-compassion, and emotion regulation

Classic psychedelics used with therapeutic intent have been shown to reduce internalized shame (79, 80) and increase self-compassion (81). Particularly noteworthy in the present study were findings that ceremonial use of ayahuasca altered longitudinal measures of self-compassion and shame. In a systematic review of empirical literature on the relationship between self-compassion and psychopathology, increased self-compassion was associated with lower levels of mental health symptoms (82), emphasizing the importance of self-compassion for developing well-being, reducing depression and anxiety, and increasing resilience to stress (83–85). Similarly, higher levels of shame are associated with lower satisfaction with mental health and characterized by more negative attitudes towards seeking professional help (86). In the present study, significant reductions in shame and increases in self-compassion accompanied reductions in depression and anxiety, suggesting potential mechanisms for the improvement of mental health and wellbeing after the ingestion of psychedelic substances. These results are consistent with prior work indicating changes in self-compassion mediate the effects of psychedelic experiences on outcomes of depression and anxiety (81).

In a prospective, longitudinal investigation of naturalistic psilocybin use, cognitive reappraisal, an aspect of emotional regulation defined as the ability to change one’s thoughts about emotionally charged stimuli, showed significant increases after psilocybin, while expressive suppression, referring to inhibition of behavioral responses to emotionally charged stimuli, showed no change. (39). Our findings are consistent with these results, indicating increases in the emotional regulation strategy of cognitive appraisal following ayahuasca use, with no change to expressive suppression. Emotion regulation strategy plays a significant part in the experience and expression of emotions and their effect on physical and mental health (87–89). Cognitive reappraisal, which denotes the ability to change one’s thoughts about emotionally charged stimuli, showed significant increases after ayahuasca ingestion, while expressive suppression, referring to inhibition of behavioral responses to emotionally charged stimuli, showed no change. Positive cognitive reappraisal strategies are correlated significantly and positively with positive indicators of mental health (90) and are generally associated with healthier patterns of social and emotional functioning than expressive suppression (91). Existing literature on positive cognitive reappraisal suggests a moderating role of cognitive reappraisal on the relationship between stressor exposure and psychopathology, presenting positive cognitive reappraisal as a viable candidate for increasing wellbeing and decreasing negative symptomology (92).

### Predicting acute psychedelic effects after ayahuasca ingestion

We observed significant associations between several presession-measures and acute post session outcomes occurring immediately after (1-3 days) the ayahuasca experience. Acute experiences of mysticism, emotional breakthrough, psychological insight, and awe were significantly associated with several presession measures, including the Psychedelic Predictor Scale (PPS), which captures thoughts and expectations right before the experience and the willingness to surrender to the experience across two subscales of set and setting (55), the Tellegen Absorption Scale (TAS), which assesses openness to altered states (52), and the State of Surrender (SoS), which is defined as a readiness to accept whatever was, whether good or bad, without resisting or fighting or struggling (38). Interestingly, the PPS was associated with all four measures of acute experiences, including mystical experiences, emotional breakthrough, psychological insight, and awe, suggesting it’s a strong predictor of multiple acute outcomes of the psychedelic experience. Consistent with prior research, our data suggests that having clear intentions and positive expectations for the psychedelic experience facilitates the occurrence of acute psychedelic experiences which may in turn lead to positive outcomes in measures of depression, anxiety, shame, self-compassion, and emotional regulation. While a relatively new scale, these results suggest the PPS may be a useful tool to measure psychedelic preparedness and predict outcomes to longitudinal changes in mental health and wellbeing, although further studies are needed to investigate whether these results are generalizable to less homogenous samples and different psychedelic substances, and a specific validation on the PPS is still pending.

### Longitudinal changes and acute psychedelic effects after ayahuasca ingestion

Findings from the current study revealed that changes in measures of depression and anxiety were significantly associated with communitas and awe scores, respectively. Communitas reflects relational experiences of perceived togetherness and shared humanity, and is associated with increases in psychological wellbeing, social connectedness, and other mental health outcomes (60). Given the shared background and homogeneity of the sample in the present study, perceived togetherness of the participants may have been an important contributing factor for positive outcomes of psychedelic ingestion. Holding safe spaces has been an essential component of community-based harm reduction in the use of psychedelics (93). A sense of belonging has been associated with decreased symptoms of depression (94), suggesting an important link between relationship-oriented experiences and depression. Awe, on the other hand, is a complex emotion comprised of an appraisal of vastness and a need for accommodation that has been associated with benefits to mental and physical health (95). Here, awe was positively associated with decreased symptoms of both state and trait anxiety. Together, we found that longitudinal changes in depression and anxiety may be mediated by acute psychedelic effects related to perceived togetherness and awe, respectively.

Consistent with prior work examining longitudinal health outcomes of psilocybin (39), no changes were found in self-reported physical health following ayahuasca use, suggesting minimal impact of ceremonial ayahuasca use on physical health factors. In contrast to Nayak and colleagues, however, we observed no changes in spiritual well-being, cognitive flexibility, or personality. Differences in the phenomenological experience of ayahuasca and psilocybin may potentially mediate these longitudinal outcomes, but further research in the general population is needed to assess this possibility.

### Longitudinal subjective changes after ingestion of ayahuasca

In the days immediately following the ayahuasca ceremony, nearly all participants rated the overall experience as positive, and most considered the experience as “Extremely Positive”. Despite many of the participants reporting that the experience was one of the most psychologically challenging experiences of their life, most considered the experience among the top 10 most personally meaningful, most spiritually meaningful, and most psychologically insightful experiences of their life. The majority of participants reported notable behavioral changes at both longitudinal follow-ups, with the most reported changes being improved relationships with others, improved diet/nutrition, improvements in career/work life, and increased physical activity/exercise. Additionally, many participants reported reduced use or craving of alcohol and other drugs consistent with prior research reporting reduced use of substances such as alcohol, tobacco, and cannabis after ayahuasca use (96–98), These findings suggest ceremonial ayahuasca may produce significant positive changes in lifestyle and behavior and hold broad benefits to general wellbeing in MENA individuals.

### Negative effects

Overall, ayahuasca use is considered to be physiologically and psychologically safe, especially in controlled settings [for a review, see (33)]. Although preliminary, the evidence presented here is consistent with these conclusions. While some participants reported both acute symptoms experienced during and immediately following (<24 hours) the ayahuasca experience, including nausea and/or vomiting, lack of appetite, restlessness, headaches, and physical pain, among others, most of these symptoms resolved in the 24 hours following the experience. It is also important to note that acts of purging, such as vomiting, are considered integral to the therapeutic use of ayahuasca and should not be dismissed as a side effect but rather reconsidered for its potential therapeutic effects (99). A minority of participants reporting persisting symptoms including lack of appetite and fatigue. However, no respondents reported seeking medical care during the experience, and only some participants reported seeking psychological care during the ceremony, including speaking with a trained therapist, helpers, and other participants on site, highlighting the importance of experienced on-site care for those attending psychedelic ceremonies. Almost all participants reported no persisting negative effects at each longitudinal follow-up, providing preliminary evidence suggesting ayahuasca use within a ceremonial setting attended by experienced facilitators represents a relatively low risk for this specific, less studied (MENA) population.

### Study limitations

The current study findings have several limitations and should be interpreted carefully. Due to the homogeneity of the majority immigrant and refugee female sample of MENA descent, as well as the small sample size of the participant group, findings of the present study should not be generalized to the wider population. Because the data were gathered online and in different settings prior to and after the ceremony, it is not possible to verify participant responses and response bias among this sample may have influenced how participants chose to answer survey questions. Additionally, a further limitation includes a lack of an experimental control group for an adequate comparison. Finally, in contrast to previous results, as such, these results should be treated as preliminary evidence requiring further investigation in controlled settings.

## Conclusion

For immigrants and refugees of MENA descent, postmigration challenges tied to resettlement in another country pose a significant risk for poor mental health and psychological wellbeing, and these challenges are particularly salient in light of limited access to health care and educational standards. There is a critical need to attend to MENA individuals’ experiences of cumulative trauma, particularly as it relates to the process of immigration or refugee resettlement. Our findings revealed ceremonial use of ayahuasca in a group of primarily female immigrants and refugees of MENA descent is associated with significant improvements in mental health, well-being, and psychological functioning, including depression, anxiety, shame, emotion regulation strategies of cognitive reappraisal, and self-compassion. Most participants reported no lasting adverse effects and experienced notable positive behavioral changes persisting months after ingestion. While preliminary, these results suggest that naturalistic ayahuasca use may hold therapeutic potential for MENA populations exposed to trauma prior to and during the process of migration.

## Data availability statement

The raw data supporting the conclusions of this article will be made available by the authors, without undue reservation.

## Ethics statement

The studies involving humans were approved by Western Institutional Review Board Copernicus Group. The studies were conducted in accordance with the local legislation and institutional requirements. The participants provided their written informed consent to participate in this study.

## Author contributions

ML: Writing – review & editing, Writing – original draft, Visualization, Validation, Supervision, Resources, Project administration, Methodology, Investigation, Funding acquisition, Formal analysis, Data curation, Conceptualization. HK: Writing – review & editing, Writing – original draft, Visualization, Validation, Methodology, Investigation, Formal analysis, Data curation, Conceptualization. DJ: Writing – review & editing, Investigation, Funding acquisition, Conceptualization. RC: Writing – review & editing, Validation, Supervision, Methodology, Investigation, Formal analysis, Conceptualization. HJ: Writing – review & editing, Validation, Supervision, Resources, Project administration, Methodology, Investigation, Funding acquisition, Conceptualization.
