# Supplementary material for: Long-term benefits to psychological health and well-being after ceremonial use of Ayahuasca in Middle Eastern and North African immigrants and refugees
Source: Front Psychiatry. 2024 Apr 10;15:1279887. doi: 10.3389/fpsyt.2024.1279887 (PMC11044680; doi:10.3389/fpsyt.2024.1279887)
Supplement: Supplementary file 1 [file Table_1.docx]

**Supplementary Materials**

**Participant Instructions**

Participants received the following instructions from the ceremony guide to prepare for the ceremony:

**CLOTHES:**

Comfortable clothes, nothing tight or constricting especially around the waist layers. Your temperature can change from cold to hot fast! So easily put on or taken off layers. Light colored clothes. White is recommended easily slipped on shoes. Clothes for the days before ceremony should be comfortable for lounging, hiking, stretching.

**OTHER:**

Refillable water bottle

Eye mask

Journal and pen

Any snacks within diet that you would like to have with you

Any items that are meaningful to you that could ground you during ceremony or that you would like to lay on the alter for ceremony (stones, pictures, symbolic items...)

Yoga mat, blankets, pillows. We will provide all items needed but feel free to bring anything that would make you feel more comfortable

Musical instruments if you would like to share songs with the circle

**SUBSTANCES TO AVOID:**

• Alcohol

• Marijuana and other drugs

• Caffeine (the best way to stop caffeine is to incrementally lower your consumption

starting today to avoid withdrawals)

• Red meat

• Pork

• Shellfish

• Refined sugars

• Nuts (on a limited basis)

• Dairy

• Fermented foods (yeast/pickles/vinegar/most salad dressings, etc)

• Soy products (including miso and tofu)

• Salty foods

• Fried Foods

• Spicy foods

• Chocolate

• Overripe fruits

• No salt or citrus on the day of ceremony only

• No sex (or masturbation) for 3 days before the ceremony (and 3 days after).

**MEDICATIONS TO AVOID**

* SSRI’s (any selective serotonin reuptake inhibitor)

* Amphetamines (meth-, dex-, amphetamine)

* Antihypertensives (high blood pressure medicine)

* Appetite Suppressants (diet pills)

* Medicine for Asthma, Bronchitis,

* Antihistamines, Medicines for Colds, Sinus Problems, Hay Fever, or Allergies

* CNS (central nervous system) Depressants

* Antipsychotics

* Antihistamines, Decongestants and Cough Medicines

* Antidepressants

**DRUGS TO AVOID**

* Actifed DM

* Amantadine hydrochloride (Symmetrel)

* Amoxapine (Asendin)

* Benadryl

* Benylin

* Bupropion (Wellbutrin)

* Buspirone (BuSpar)

* Carbamazepine (Tegretol, Epitol)

* Chlor-Trimeton

* Clomipramine (Anafranil)

* Cocaine Compoz

* Compoz

* Cyclobenzaprine (Flexeril)

* Cyclizine (Marezine)

* Desipramine (Pertofrane)

* Dextromethorphan (DXM)

* Disopyramide (Norpace)

* Doxepin (Sinequan)

* Ephedrine

* Flavoxate Hydrochloride (Urispas)

* Fluoxetine (Prozac)

* Imipramine (Tofranil)

* Isocarboxazid (Marplan)

* Levodopa (Dopar, Larodopa)

* Loratadine (Claritin)

* Maprotiline (Ludiomil)

* Meperidine (Demerol)

* Methylphenidate (Ritalin)

* Nortriptyline (Aventyl)

* Oxybutynin chloride (Ditropan)

* Orphenadrine (Norflex)

* Parnate

* Paroxetine (Paxil)

* Phenergen

* Phenelzine (Nardil)

* Procainamide (Pronestyl)

* Protriptyline (Vivactil)

* Pseudoephedrine

* Quinidine (Quinidex)

* Salbutemol

* Salmeterol

* Selegiline (Eldepryl)

* Sertraline (Zoloft)

* Tegretol

* Temaril

* Tranylcypromine (Parnate)

* Tricyclic antidepressants (Amitriptyline, Elavil)

* Trimipramine (Surmontil)

* Yohimbine

**Assessing Significance with Linear Mixed Models**

To assess overall significance of primary and secondary outcomes, linear mixed models with the fixed effect of Time (baseline; 2-4 weeks post-session; 3-4 months post-session) were performed.

***Mood.*** A linear mixed model with the fixed effect of Time (baseline; 2-4 weeks post-session; 3-4 months post-session) was significant (p = 0.006; F = 6.38).

***Anxiety****.* A linear mixed model with the fixed effect of Time (baseline; 2-4 weeks post-session; 3-4 months post-session) was significant for state (p = 0.040; F = 3.58) but not trait (p = 0.147; F = 2.05) anxiety.

***Emotion Regulation.*** A linear mixed model with the fixed effect of Time (baseline; 2-4 weeks post-session; 3-4 months post-session) was not found to be significant for either reappraisal (p = 0.082; F = 2.77) or expressive suppression (p = 0.401; F = 0.95).

***Self-compassion.*** A linear mixed model with the fixed effect of Time (baseline; 2-4 weeks post-session; 3-4 months post-session) was significant (p = 0.037; F = 3.78).

***Shame.*** A linear mixed model with the fixed effect of Time (baseline; 2-4 weeks post-session; 3-4 months post-session) was significant (p = 0.032; F = 4.00), warranting further investigation.
